# The National Scheme

**Published:** 1920-06-05

**Authors:** 


THE PUBLIC WELL-BEING—(*continued*).

### The National Scheme.

### General Lines of Criticism and Appreciation.

The report of the Medical Consultative Council, in its outline of a new national health system, has attracted prominent attention in the lay Press, and, generally speaking, has been favourably received, though in nearly every instance some alarm has been expressed at the great cost which seems to be involved if it is carried out *en bloc*. Most papers, however, assume that the whole structure will be built up gradually.

*The Times* says the first and most obvious criticism of the scheme is that it is likely to prove very expensive. The idea of establishing secondary health centres—*i.e.*, hospitals—at the present time may be ruled out on the ground of cost, though later this enterprise may become feasible. The primary centres, however, are much more important. They could be opened at a very small cost, as has been shown by the experiments of Sir James Mackenzie at St. Andrews. By adding at once to the working power of the general practitioner they must serve to relieve the strain on all hospitals. Moreover, in these centres the general practitioners will be able to begin their research work on the early stages of disease. The policy of enabling disease to be recognised early and so cured will rapidly repay its very small cost, for the need for large hospitals will grow smaller and the price to be paid for chronic diseases will be reduced. The idea that the general practitioner should be able to become a consultant is an excellent one.

### Practical Ideal.

The *Morning Post* says that the Consultative Council has produced an ideal scheme, but admittedly it is impossible to carry out immediately the recommendations as a whole. A new Act of Parliament will be required, and the universal application of the scheme would mean a staggering demand on the Treasury. It is a comprehensive scheme for future realisation, and represents fairly the medical ideal of simplification and symmetry in the conduct of professional services in the public interest. The Council is to be congratulated in drawing a practical picture of the medical aspirations to work with scientific freedom for the public weal. Those who would reproach it because it is impossible to put the scheme into immediate working must not forget that it has been deliberately furnished as an ideal which should be tested in favourable centres before it can be applied in a general manner. This is practical idealism.

### Leadership of the World.

The *Daily Telegraph* considers that the report foreshadows a very remarkable development in the national health policy, and from every point of view the organised services proposed would be a vast improvement on what is possible under the existing conditions. The essence of the scheme has been under professional discussion long enough to deprive it of any startling novelty to medical men. It has already been actually adopted and worked out by the public authorities and the medical profession in Gloucestershire, thanks to the energy and enthusiasm of the Medical Officer of Health, Dr. J. Middleton Martin, upon whose detailed scheme the proposals of the report are based, and there has been experimental action in th same direction elsewhere. It is plainly out of the question, however, in the present state of the national finances, to establish the whole of the organisations proposed at one stroke, but the establishment of the primary centres, which is the heart of the scheme, may be found feasible, for patients who now pay for medical treatment will be required to do so still, while more effective treatment of all diseases and the better organisation of preventive methods would lead to the saving of a mass of expenditure now incurred in connection with public health. Whatever form the Government legislation may take, it will at any rate be such as to show this country to be in the leadership of the world, as has long been the case where matters of public health are concerned.

### State Medical Service.

The *Daily News* is rather critical, and says the scheme merely promises to add to medical buildings and systems without any promise of wider distribution of medical treatment. So far as they would remove the charity from hospitals and make it easy for patients to go there and pay for value received, the proposed centres would open up a fruitful reform. It criticises the rejection of the idea of a State medical service, and says that schemes drawn up in antagonism to this idea are so much potential rubbish which the medical statesmen of the future will have to clear away.

As may, of course, be expected the *Daily Herald* also harps upon this theme, and glibly propounds the loose doctrine that until wealth is commonly owned there can be no common enjoyment of health.

The *Manchester Guardian* says that in the comprehensive treatment of public health at its root lies the real strength of the scheme, which is marked by imagination and thoroughness. It expresses the view, however, that many laymen will disagree with the rejection of the whole-time salary for medical services, but they will not, therefore, look with disfavour upon the plan, for the main benefits which will accrue from such a service are secure. If any approach to unanimity on the value of such services be reached, the change can be made with much greater ease from the basis of this plan than from present conditions. It is sufficient for the moment to merge the work done by educational authorities, hospitals, child-welfare centres, and many other bodies in a common centre, and to relate to that centre a general practitioner. This much the plan would achieve, and criticism should recognise the boldness of this central conception and view it in no sectarian spirit.

### Logical But Bureaucratic.

The *Yorkshire Post* considers the scheme gives a great opening for a new bureaucracy intimately affecting the life of every individual, and finds reason for the fear in the statement of Sir Berkeley Moynihan that 50 or 60 per cent. of the million men incapable of being put in any medical category under the Conscription Act would have been in perfect health if there had been adequate supervision over their previous life. The scheme, however, is admirably logical, and one of its good features is that it can be adopted piece-meal. It advocates the absorption of Poor-Law infirmaries and voluntary hospitals, which are to be "encouraged" to fall into line as part of the secondary centres—the "encouragement" to take the form of withholding grants from those which remain outside. Voluntary hospitals would find it difficult to stand out against such persuasion. Yet there will be much sympathy with opposition to the surrender of that

**THE PUBLIC WELL-BEING**—(*continued*)

independence which coincides with, if it has not largely helped to, the very advance of science which is the main reason put forward for the Council's scheme. This scheme has features which will arouse much opposition, but also many excellent details, amongst the best being the provision for enlarging the supply of doctors, nurses, and dentists.

The *Birmingham Post* regards the report as "the thoughtful idealism of professional men," who have "aimed at a neatness and precision of place not as a rule associated with British institutions." On paper, this paper proceeds, the plan looks impressive; but it criticises the financial side of the matter, or rather the omission to consider it by the Consultative Council. It at least "agrees that the scheme is vastly better than any plan involving the handing over of the public to a State-controlled, whole-time medical service."

So far as the Press publishes individual views of medical men, these views are broadly favourable to the report.

